# Sonic Hedgehog in SCLC

**DOI:** 10.18632/aging.100817

**Published:** 2015-09-26

**Authors:** Mikko O. Laukkanen, J Silvio Gutkind, Maria Domenica Castellone

**Affiliations:** Istituto di Endocrinologia ed Oncologia Sperimentale “G. Salvatore” (IEOS), C.N.R. 80131- Naples; Italy

**Keywords:** Sonic Hedgehog, SCLC, BN/GRPR

The Hedgehog (Hh) signal transduction pathway has been discovered as a central regulator of embryonic development, tissue maintenance and repair [1]. Moreover, several recent evidences have highlighted its key function in tumorigenesis [2]. In some familial cancers, such as basal cell carcinoma and medulloblastoma, Hh pathway activation represents the initial tumorigenic event, whereas in other human malignancies, including gastrointestinal, lung, brain, breast and prostate cancers, deregulation of Hh signaling occurs during tumor progression and participates in tumor maintenance. Clinical trials using molecular inhibitors targeting Hh pathway components, in particular the Smo receptor, have often yielded limited clinical benefits unless they are used for the treatment of tumors harboring defined genetic mutations inactivating tumor suppressors (e.g. Ptch receptor) or activating oncogenes (e.g. Smo receptor, Gli transcription factor, Shh ligand) within the Hh pathway. In these specific cases, promising results led to FDA approval of Vismodegig (GDC0449, Genentech), a Smo inhibitor, in the treatment of basal cell carcinoma and medulloblastoma [3]. Notwithstanding, several clinical studies using the same compound in tumors exhibiting Hh overactivity without identified Hh mutations have resulted in discouraging outcome and discontinuation of the trials because of lack of objective response [2]. These unexpected results have been related to drug resistance due to the presence of activating mutations or genetic alterations driving Hh signaling bypassing Smo function. These evidences emphasize the need for further characterization of Hh signaling in tumorigenesis and for a more precise identification of the interaction between Hh and other signaling pathways involved in tumor development and response to therapy.

Small cell lung carcinoma (SCLC) is a very aggressive cancer with extremely poor prognosis, whose genetic events, such as oncogenic driver mutations, have not been defined yet. Classified as neuroendocrine tumors, SCLCs secrete factors of the bombesin (BN)/Gastrin-Releasing Peptide (GRP) family and express their cognate receptors activating an autocrine loop that increases proliferation and survival [4]. The positivity for this ligand/receptor pair is considered to be a marker of aggressiveness and unfavorable tumor outcome. Recent reports have described the Hh pathway as a key regulator of lung embryogenesis and SCLCs maintenance, although no mutations in Hh signal transduction pathway molecules have been identified, suggesting a ligand-dependent pathway activation [5, 6]. The ligand-dependent activation of Hh signaling can occur in an autocrine manner, where cancer cells express both the ligand and the receptor, or in a paracrine manner, where ligand produced from cancer cells is activating Hh signaling in tumor stroma or vice versa.

To characterize Hh function in SCLC and to evaluate the therapeutic potential of Hh inhibitors in this cancer, we have investigated the possibility of a direct interaction between Hh and BN/GRPR signaling pathways. According to our initial observations, Cyclopamine, an inhibitor of Smo, attenuated BN induced cell proliferation. In support of these data, RNA interference for Sonic Hedgehog (Shh), upstream activator of Smo, reduced BN stimulated growth, matrigel spreading and soft agar colony formation [7]. Surprisingly, when testing the activation of Gli transcription factor upon BN stimulation, we revealed the existence of a direct crosstalk between the two pathways. In order to dissect the signaling molecular events mediating this interaction, we discovered that BN, through its G protein coupled receptor (GRPR) linked to Gαq/Gα12/13 large G proteins, and their downstream target, the Rho small GTPase, was able to stimulate NFkB-mediated transcription of Shh, thus initiating an autocrine signaling loop that links BN/GRPR pathway to production of Shh ligand, and the autocrine Hh signaling activation (Figure 1, left panel) [7].

**Figure:**
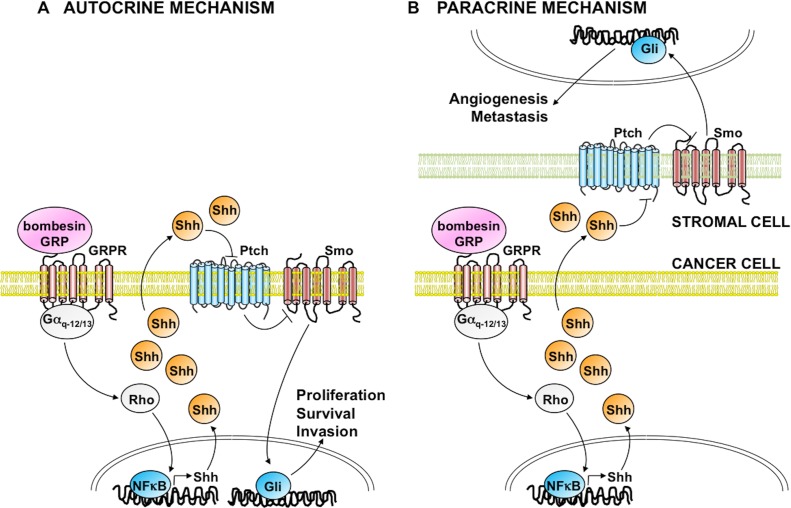


Our findings, besides shedding new light on the mechanisms of Hh signaling activation in SCLC, may suggest a more general application for other BN/GRPR positive tumors over-expressing Shh pathway, such as pancreatic cancer, neuroblastomas and glioblastomas. Interestingly, use of Hh inhibitors alone in these tumors has not reached positive results that may indicate existence of functional parallel pathways able to counteract the effect of the drug. Moreover, recent reports have highlighted a role for Hh signaling in tumor-stroma interactions, with production of Shh ligand from cancer cells and stimulation of Gli transcription factor in tumor microenvironment (myofibroblasts, endothelial cells, and CSC) [8]. In our study, we have investigated the existence of an autocrine ligand-dependent Hh signaling in SCLC. We certainly believe that it would be interesting to study also the paracrine activation of Hh signaling, which could have the double effect of stimulating proliferation and survival of stroma cells, leading to increased angiogenesis and metastasis and, at the same time, produce growth factors acting on cancer cells to sustain their proliferation, epithelial-to-mesenchymal transition (EMT), dissemination and survival (Figure 1, right panel). Our data connecting BN/GRPR and the Hh signaling pathway may therefore provide valuable knowledge on the complex interaction between tumor cells and cancer microenvironment and may offer the scientific basis for developing novel therapeutic stategies that, by combining different anti-tumor approaches, could be more effective than single agent treatments. In this case, novel co-targeting strategies would target not only cancer cells but also other component of tumor microenvironment. Moreover, simultaneous targeting of BN/GRPR and Hh pathway could help in counteracting mechanisms of cell-autonomous and non-cell autonomous (stroma-dependent) resistance to targeted therapies.
